# Age Effect on Automatic Inhibitory Function of the Somatosensory and Motor Cortex: An MEG Study

**DOI:** 10.3389/fnagi.2018.00053

**Published:** 2018-03-02

**Authors:** Chia-Hsiung Cheng, Mei-Yin Lin, Shiou-Han Yang

**Affiliations:** ^1^Department of Occupational Therapy and Graduate Institute of Behavioral Sciences, Chang Gung University, Taoyuan, Taiwan; ^2^Laboratory of Brain Imaging and Neural Dynamics (BIND Lab), Chang Gung University, Taoyuan, Taiwan; ^3^Healthy Aging Research Center, Chang Gung University, Taoyuan, Taiwan; ^4^Department of Psychiatry, Chang Gung Memorial Hospital, Linkou, Taiwan

**Keywords:** beta rebound, somatosensory gating, inhibition, aging, magnetoencephalography (MEG)

## Abstract

Age-related deficiency in the top-down modulation of cognitive inhibition has been extensively documented, whereas the effects of age on a bottom-up or automatic operation of inhibitory function were less investigated. It is unknown that whether the older adults (OA)’ reduced behavioral performance and neural responses are due to the insufficient bottom-up processes. Compared to behavioral assessments which have been widely used to examine the top-down control of response inhibition, electrophysiological recordings are more suitable to probe the early-stage processes of automatic inhibitory function. Sensory gating (SG), a phenomenon of attenuated neural response to the second identical stimulus in a paired-pulse paradigm, is an indicator to assess automatic inhibitory function of the sensory cortex. On the other hand, electricity-induced beta rebound oscillation in a single-pulse paradigm reflects cortical inhibition of the motor cortex. From the neurophysiological perspective, SG and beta rebound oscillation are replicable indicators to examine the automatic inhibitory function of human sensorimotor cortices. Thus, the present study aimed to use a whole-head magnetoencephalography (MEG) to investigate the age-related alterations of SG function in the primary somatosensory cortex (SI) and of beta rebound oscillation in the primary motor cortex (MI) in 17 healthy younger and 15 older adults. The Stimulus 2/Stimulus 1 (S2/S1) amplitude ratio in response to the paired-pulse electrical stimulation to the left median nerve was used to evaluate the automatic inhibitory function of SI, and the beta rebound response in the single-pulse paradigm was used to evaluate the automatic inhibitory function of MI. Although there were no significant age-related differences found in the SI SG ratios, the MI beta rebound power was reduced and peak latency was prolonged in the OA. Furthermore, significant association between the SI SG ratio and the MI beta rebound power, which was seen in the younger adults (YA), was absent in the OA. In conclusion, our data suggested an age-related defect of association between sensorimotor cortices regarding automatic inhibitory function.

## Introduction

It has been well documented that physiological aging is associated with reductions in various domains of cognitive abilities, such as working memory, processing speed, attention and inhibitory function (Hedden and Gabrieli, 2004; Cheng and Lin, 2012; Cheng et al., 2012). Among these domains, inhibition is of particular importance since efficient performance of goal-directed tasks requires not only selective attention to task-relevant information, but also effective inhibition in task-irrelevant stimuli. Inhibitory function comprises a top-down mechanism that allows for the manipulation of stimulus information, as well as a more bottom-up mechanism that is operated to automatically react to sensory inputs (Fritz et al., 2007; Lijffijt et al., 2009; Liu et al., 2011; Cheng et al., 2016b). Accumulated evidence has revealed an age-related deficiency in the top-down modulation of inhibitory control measured by the Go/Nogo (Vallesi et al., 2009; Vallesi, 2011; Mudar et al., 2015; Kropotov et al., 2016) and stop-signal (Bloemendaal et al., 2016; Coxon et al., 2016) paradigms. However, the findings regarding effects of age on automatic inhibitory function is relatively limited and inconsistent.

Sensory gating (SG) is conceptualized as an ability of the brain to automatically filter out irrelevant information to protect the higher-order brain centers from sensory inundation (Boutros and Belger, 1999; Light and Braff, 2000; Potter et al., 2006; Wan et al., 2008). Paired-pulse auditory stimulation is a well-known paradigm that evaluates SG in healthy subjects and even patients with neurological and psychiatric disorders (Ambrosini et al., 2001; Patterson et al., 2008; Cheng et al., 2016a). Accumulating evidence has shown that pre-attentive SG is associated with many aspects of cognitive function, such as working memory, attention and processing speed, in these clinical patients (Smith et al., 2010; Thomas et al., 2010). SG can be induced by a paired-stimulus paradigm in which two consecutive stimuli are presented with an inter-stimulus interval (ISI) of 500 ms and an inter-pair interval of more than 6 s (Boutros and Belger, 1999). Quantitatively, SG is calculated by the amplitude ratio of Stimulus 2 over Stimulus 1 (S2/S1) and a lower S2/S1 ratio is indicative of better SG function (Boutros and Belger, 1999; Cheng et al., 2015a). Besides the auditory modality, SG can also be tested with electrical stimulation (Stevenson et al., 2012; Wiesman et al., 2017). Although a previous event-related potential (ERP) study reported a reduced SG function in the human primary somatosensory cortex (SI) as a function of age (Lenz et al., 2012), our event-related field (ERF) studies did not observe significant SG differences in the SI between younger and older adults (YA and OA; Cheng and Lin, 2013; Cheng et al., 2015b). Due to the above controversial results, we were motivated to capitalize magnetoencephalography (MEG) to perform a further in-depth investigation of SI SG. Compared to electroencephalography (EEG), MEG has not only equally excellent temporal resolution but also superior spatial resolution, allowing us to elucidate the automatic inhibitory function at the cortical level. Furthermore, the experiment design is equivalent in both EEG and MEG recordings, which enables us to reconcile previous results by using the same stimulus parameters. Taken together, the first aim of the present study was to investigate the effects of age on the SI SG by means of MEG with the analysis at the cortical level.

In addition to the time-locked evoked responses, electrical stimulation to the median nerve also elicits non-time-locked brain rhythmic activities. The beta oscillations (13–30 Hz) initially were suppressed and then rebound prominently at 400–900 ms after the stimulus onset (Hari et al., 1998; Tominaga et al., 2009). Several lines of evidence have indicated that this post-stimulus beta rebound originates in the precentral motor cortex (MI), and is associated with increased motor cortical inhibition (Pfurtscheller et al., 1996; Cassim et al., 2001; Gaetz and Cheyne, 2006). For example, it has been evident that the beta rebound of MI is clearly observed after the electrical stimulation when the subjects’ hands are at the resting position. However, this rhythmic activity is partially decreased during the observation of other’s hand movements, and even more suppressed when they actively perform voluntary movements (Hari et al., 1998; Järveläinen et al., 2004; Cheng et al., 2017a). Thus, a more suppressed beta rebound reflects a more excitation of MI; on the other hand, a higher level of this induced rhythmic component indicates an increased motor cortical inhibition (Pfurtscheller et al., 1996; Gaetz and Cheyne, 2006). Because subjects’ voluntary responses to the stimulation are not required in the experiment, the beta rebound oscillation is considered to be an indicator of automatic, bottom-up inhibitory function in the MI. For example, patients with cerebral palsy (Pihko et al., 2014) or complex regional pain syndrome (Kirveskari et al., 2010) have shown decreased stimulus-induced beta rebound activities when their hands were in the resting condition, suggesting that a higher level of this oscillation is indicative of a more efficient processing of cortical inhibition. In the OA, when subjects’ ankles were passively moved without voluntary motor response, Toledo et al. (2016) observed a significant reduction of beta oscillatory power in the OA than in YA. However, up to this day, there is no empirical study investigating the effects of age on the MI beta activities, both for amplitude and latency, induced by electrical stimulation on the median nerve. Therefore, the second aim of the present study was to examine the age-related differences in the electricity-induced beta-frequency dynamics of the MI.

Regarding the connectivity between SI and MI, previous anatomical studies have shown rich fiber connections between these two regions (Pavlides et al., 1993; Shinoura et al., 2005; Petrof et al., 2015). These findings provide empirical support to the notion that coordinated movements substantially depend on precise feedbacks of somatosensory information (Lin et al., 1993; Brochier et al., 1999). Recent functional MRI studies have demonstrated that greater resting-state functional connectivity in the sensorimotor network was associated with better motor performance in the OA (Seidler et al., 2015; He et al., 2017). Another study also indicated that the changes in the resting-state of sensorimotor system could be used to estimate the age of OA (Qiu et al., 2015). However, most of these results were derived from the fMRI “resting-state” data, and it remains elusive whether the relationship between SI (as indexed by somatosensory SG) and MI (as indexed by beta rebound activities) in terms of “automatic inhibitory function” is also altered due to aging. Hence, the third aim of the present study was to elucidate the association between SI SG and MI beta rebound in the YA and OA and compare the strength of this association between these two groups.

More specifically, we applied paired-pulse and single-pulse electrical stimulation to study SI SG and MI beta rebound activities, respectively. The neuromagnetic responses were recorded with a 306-channel whole-head MEG and the data were analyzed with the distributed source modeling of depth-weighted minimum norm estimate (wMNE). Based on the previous literature review, the goals of the present study were 3-fold. First, we attempted to test whether the SI SG ratio would be higher in the OA than in the YA. Second, we examined whether the OA would have lower amplitude of the MI beta rebound oscillations. Due to no prior work addressing the latency of MI beta rebound activities, we also sought to explore the age-related differences in the peak latency of MI beta rebound oscillations. Third, and most importantly, we examined whether the association of automatic inhibitory functions between SI and MI would be reduced in the aged adults.

## Materials and Methods

### Participants

We recruited 17 healthy YA (aged 20–34 years old; four females), and 15 healthy OA (aged 60–74 years old; five females) in the present study. All the subjects were collected through word-of-mouth and online bulletin board advertisements, and most of them were from an existing magnetic resonance spectroscopy (MRS) database (Cheng et al., 2017b). All participants were right-handed and self-reportedly with no history of neurological or psychiatric disorders. Individuals in the OA group were also administered the Cognitive Ability Screening Instrument (CASI; Lin et al., 2002), and a score lower than 79 out of 100 was indicative of mild cognitive impairments. The mean (M) ± standard error of the mean (SEM) of the OA was 90.8 ± 1.47, which indicated that they did not suffer from cognitive impairments. All procedures were approved by the Institutional Review Board of Taipei Veterans General Hospital (Taipei, Taiwan), and were performed in accordance with approved guidelines and regulations. A written informed consent was obtained from each participant.

Some data were excluded since the beta rebound oscillations could not be detected in four subjects of the YA group. Thus, a total of 13 younger and 15 older participants were included in the final comparisons of amplitude and latency of MI beta rebound and correlation analysis.

### Procedures and Stimuli

MEG recordings consisted of two blocks with a counterbalanced sequence. The paired-stimulus paradigm was designed to study SI SG function, and the single-stimulus paradigm was used to measure MI beta rebound oscillations. In the paired-stimulus paradigm, the left median nerve was stimulated twice consecutively, with an ISI of 500 ms and an inter-pair interval of 6 s. In the single-stimulus paradigm, electrical stimuli were delivered repeatedly with an ISI of 1.6–2.0 s. All the stimuli were given with 0.2-ms constant-current square-wave pulses by an electrical stimulator (Konstant-Strom Stimulator, Schwind, Erlangen, Germany). Stimulus intensity was set at 20% above the motor threshold of each individual to elicit an obvious twitch of the thumb. A wet belt tied on the upper arm was used as the ground because this strategy could substantially reduce the electricity-induced artifacts. During the MEG recordings, the subjects were instructed to watch a self-chosen silent movie and to ignore the experimental stimuli. The movies were emotionally neutral so that they would not have caused overt reactivity of the affective system in the individual.

### MEG Recordings and Analyses

The neuromagnetic responses to electrical stimulation were continuously recorded by a 306-channel MEG (Vectorview, Elekta Neuromag, Helsinki, Finland), with a sampling rate of 1000 Hz and an online bandpass of 0.1–200 Hz. The data from 204 planar gradiometers, which detect the largest signals directly above the activated cortical regions, were analyzed. At least 100 artifact-free trials in each paradigm were collected.

The modeling of cortical spatiotemporal dynamics of neuromagnetic responses was performed with Brainstorm (Tadel et al., 2011). The segmentation of head tissues from each individual’s structural images (TRIO SIEMENS 3T MR system with TR/TE-FA = 2530 ms/3.03 ms/7 degrees) was obtain from BrainVisa^1^. The representation of folded cortical surface was used to resolve the forward problem by using an overlapping-sphere model, which yields the strength of the electrical dipoles at the cortical surface (Huang et al., 1999). We applied a cortically-constrained, wMNE source modeling to reconstruct neural generators by using Brainstorm software (Tadel et al., 2011). A set of ~15,000 elementary dipoles over the cortical envelope of each participant was geometrically registered to the Montreal Neurological Institute (MNI) brain template (Colin27).

The first cortical response to the electrical stimulation is M20, followed by M35 in the SI. In the paired-pulse paradigm, there is no obvious gating effect on M20 with an ISI of 500 ms, while a significant SG is found in the M35 component (Stevenson et al., 2012; Cheng and Lin, 2013). Therefore, the M35 component was selected to study the somatosensory SG in the present study. Each subject’s largest wMNE cortical activation of M35, a cluster of 30 vertices corresponding to 4–5 cm^2^, was manually identified as region of interest (ROI) in the SI. Although the locations of maximal cortical response were a little different across subjects, the ROIs of S1 and S2 within the subject were exactly the same. For both S1 and S2, the magnitude of each dipole was then normalized to its fluctuation over the baseline (100 ms before the stimulus onset), yielding a *z*-score for each cortical location. The *z* values of M35 responses to S1 and S2 were extracted to calculate the SG ratio (S2/S1).

For the single-stimulus paradigm, the aforementioned wMNE methods were also applied to reconstruct source activation. We manually identified a set of 30 vertices corresponding to 4–5 cm^2^ in the precentral region, anterior to the selected ROI of M35, to examine the electricity-induced beta rebound activities (Gaetz and Cheyne, 2006). In order to characterize the profile of the power spectrum from each subject, the MI source waveform of each raw trial (100 ms before and 1000 after the stimulus onset) was computed by means of Morlet wavelet-based time-frequency approach, with a central frequency of 1 Hz and a time resolution of 3 s. The resolution was given in units of Full Width Half Maximum (FWHM) of the Gaussian kernel in both time and frequency^2^. The power values after *z*-normalization were computed in each participant. Although the resolution of the frequency was 1 Hz in our present study, we decided to use the average of the two largest frequency values to represent one’s beta rebound activity. More specifically, the mean strength of the most reactive beta frequencies (2 Hz) was identified and then calculated from the average of 200 ms centering the peak latency of beta rebound activities (100 ms before and 100 ms after the peak).

### Statistical Analyses

Statistical analyses were carried out with the IBM SPSS software (version 19). All the data were present with M ± SEM. The normality of each dataset (e.g., amplitude of S1 and S2, SI SG ratio, latency and amplitude of MI beta rebound) in the YA and OA groups was evaluated by Shapiro-Wilk tests, and the results showed that more than 80% of the data were not normally-distributed. Thus, we conservatively applied the non-parametric analysis in the present study. The amplitude differences between S1 and S2 in each group were compared by Wilcoxon signed rank test. The effects of age on stimulation intensity, SI SG ratio, MI beta rebound strength and latency were assessed by the Mann-Whitney U test. The associations between the SI SG ratio and the MI beta rebound power as well as latency in each group were evaluated with the Spearman’s rank correlation coefficient. Moreover, in the correlation analysis, SI SG ratio was compared twice with MI beta rebound parameters in each group, and thus a correction for multiple comparisons by the Benjamini and Hochberg approach was performed (Benjamini and Hochberg, 1995). *P* values less than 0.05 were set as the significant threshold. Effect sizes (ES) of each comparison were also reported.

## Results

**Figure 1:**
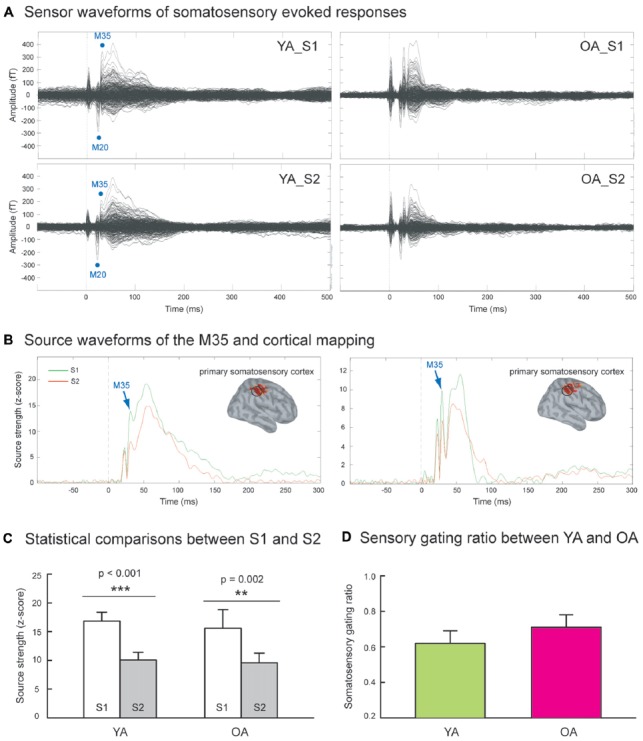
**(A)** Grand-averaged somatosensory evoked fields following paired-pulse electrical stimulation (S1 and S2) in the younger adults (YA, *n* = 17) and older adults (OA, *n* = 15). **(B)** The M35 activation of the depth-weighted minimum norm estimate (wMNE) located in the postcentral cortex was analyzed. Compared to the S1 (green traces), the M35 source amplitudes of S2 (red traces) were significantly reduced in both YA and OA. **(C)** The statistical results confirmed that the suppression of S2 was robust in both groups. **(D)** The comparisons of somatosensory gating ratio (S2/S1), however, did not show between-group differences.

Figure 1A shows the grand-averaged sensor waveforms of somatosensory evoked responses to S1 and S2 in YA and OA groups. Figure 1B displays the wMNE source activation of SI as a function of time in each group. The M35 component was reconstructed onto the postcentral wall of the central sulcus across all subjects. Figure 1C exhibits that compared to the S1, the source strength of the S2 was significantly suppressed in the YA (*p* < 0.001, one-tailed, ES = 0.80) and OA (*p* = 0.002, one-tailed, ES = 0.78) groups. In contrast to our hypothesis, Figure 1D shows no significant differences on the SI SG ratios between YA (0.62 ± 0.07) and OA (0.71 ± 0.07; *p* = 0.102, one-tailed, ES = 0.22).

**Figure 2:**
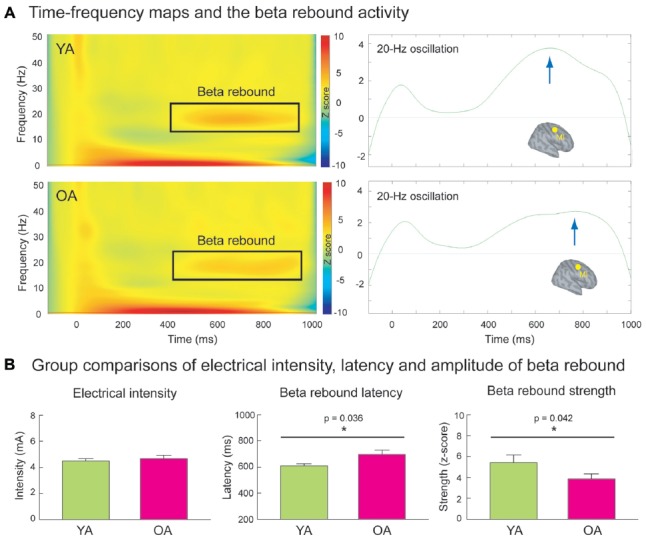
**(A)** The left panel demonstrates the time-frequency maps of electricity-induced beta rebound oscillations (black rectangles) in the YA (*n* = 13) and OA (*n* = 15). The right panel exhibits the time course of the beta rebound activities in the most reactive frequency bands (2 Hz) with respect to the baseline power in the primary motor cortex (MI). The blue arrows indicate the peak latencies of beta rebound activities. **(B)** The intensity of the electrical stimulation was equivalent between YA and OA. However, compared to the YA, the OA showed a delayed latency and reduced mean amplitude of the beta rebound activities.

Figure 2A shows the grand-averaged time-frequency maps and the beta rebound oscillations of MI in YA and OA groups. The beta rebound activities peaked at around 600–800 ms after the stimulus onset, and were reliably detected in most of the participants (Supplementary Figure S1). As shown in Figure 2B, the intensity of electrical stimulation (i.e., 20% above the motor threshold of each individual) was similar between YA (4.48 ± 0.17 mA) and OA (4.65 ± 0.26 mA; *p* = 0.910, two-tailed, ES = 0.02), suggesting no prominent peripheral effects of age on central cortical processes. However, consistent with our hypothesis, the mean strength of MI beta rebound oscillations was reduced in the OA (3.87 ± 0.47) than in the YA (5.41 ± 0.74; *p* = 0.042, one-tailed, ES = 0.33). Furthermore, compared to YA (607 ± 18.4 ms), OA (697 ± 31.5 ms) demonstrated a prolonged peak latency of MI beta rebound oscillations (*p* = 0.036, two-tailed, ES = 0.40).

**Figure 3:**
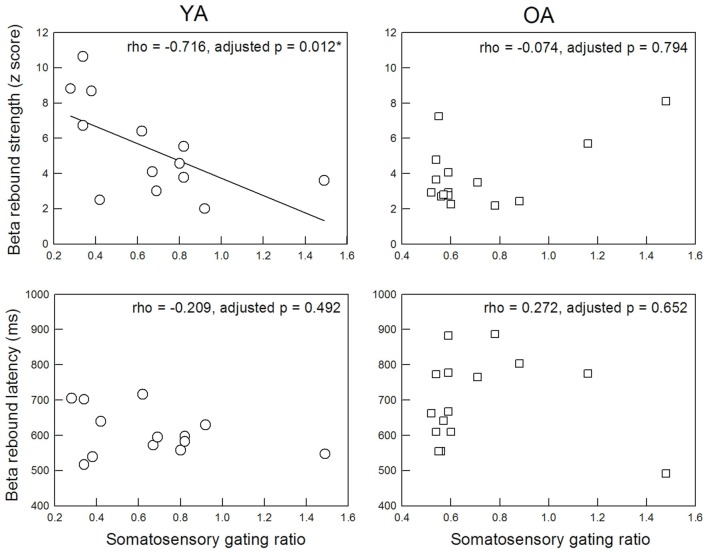
Correlation between M35 gating ratio in the primary somatosensory cortex and beta rebound activities in the MI.

Figure 3 displays the functional relationship between SI SG ratio and MI beta rebound activities. The statistical results revealed a significant association between SI SG and MI beta rebound strength in the YA group (rho = −0.716, adjusted *p* = 0.012, two-tailed, ES = 0.51), indicating a better SI SG function was correlated with a higher motor cortical inhibition. However, such association disappeared in the OA group (rho = −0.074, adjusted *p* = 0.794, two-tailed, ES = 0.005). After applying the Fisher r-to-z transformation, the correlation coefficient values between YA and OA were significantly different (*Z* = −2.5, *p* = 0.012, two-tailed). There were no significant correlations between SI SG ratio and MI beta rebound latency in either YA (rho = −0.209, adjusted *p* = 0.492, two-tailed, ES = 0.04) or OA (rho = 0.272, adjusted *p* = 0.652, two-tailed, ES = 0.07).

## Discussion

This study investigated the effects of age on the automatic inhibitory function in the SI and MI by means of MEG recordings. Although there were no significant age-related differences for the SI SG ratio, the MI beta rebound activities were lower in the OA. In addition, the association between SI SG ratio and MI beta rebound power, which was obviously seen in the YA, appeared to be diminished in the OA.

SG is considered as a common electrophysiological measure to evaluate automatic inhibitory functions. In addition to the auditory cortex, the investigation of SG in the somatosensory cortex has gained more attention in recent years and has been applied in many clinical investigations, such as dystonia (Frasson et al., 2001), cerebral palsy (Kurz et al., 2017), and multiple sclerosis (Arpin et al., 2017). In the studies of aging, a previous ERP report indicated that somatosensory SG ratio was increased as a function of age (17–86 years old), suggesting an age-related defect of automatic inhibitory function (Lenz et al., 2012). These findings were in contrast to our recent ERF data, showing that there was no obvious age-related reduction of SI SG (Cheng and Lin, 2013; Cheng et al., 2015b). However, it should be noted that, in that ERP study, the intensity of electrical stimulation was significantly larger in the older than younger group, and thus the between-group differences on SG ratio might be partially confounded by this peripheral factor. In order to reconcile the previous controversial results, our present study recruited another sample of cognitively-intact OA and the intensity of electrical stimulation was not significantly different between these two groups. Similarly, we did not find a significant reduction of SI SG in the OA group compared to the YA group. Furthermore, the present wMNE results were consistent with our previous findings by means of equivalent current dipole (ECD) modeling (Cheng and Lin, 2013), suggesting that our current findings were supported by different analytic methods. Nevertheless, we did not exclude the differential recording sensitivities to neural responses between EEG and MEG, giving rise to the controversial results. Typically, MEG sensors are very sensitive to tangential dipoles (e.g., the M35 generators of the SI), while EEG arrays collect both tangential and radial dipoles. Also, the previous ERP study used peak-to-peak (N20-P25) amplitudes to calculate S2/S1 ratios (Lenz et al., 2012), while we used baseline-to-peak amplitudes (absolute values of M35 strength) for subsequent analysis. The different strategies for amplitude measurements might also contribute to the discrepancies among these studies. Taken the present results with our series of studies together (Cheng and Lin, 2013; Cheng et al., 2015b), the SI SG function, particularly the M35 component, is relatively preserved in the cognitively-intact OA.

Over the past decades, beta oscillations were widely studied through hand or finger movements, called post-movement beta rebound (PMBR). It has been shown that PMBR after the movement was significantly reduced in the older than in the younger participants (Labyt et al., 2003). However, the design of voluntary movements, which required sustained attention and high degree of cooperation, is not suitable for the OA and those with clinical disorders. It is interesting to note that electrical stimulation to the median nerve also induces beta rebound oscillations. Several lines of evidence have validated that this electricity-induced activity is originated in the precentral cortex, i.e., MI. Compared to the YA, the elderly showed reduced MI beta rebound activities. The rationale of the relationship between electricity-induced beta oscillations and increased cortical inhibition in MI has been supported by a transcranial magnetic stimulation (TMS) study, in which the amplitudes of motor evoked potentials (MEPs) were evaluated at the conditioning-test (CT) intervals of 400, 600 and 1000 ms (Chen et al., 1999). The results showed that the interval with maximal reduction of MEP, i.e., 600 ms, corresponded to the peak latency of beta rebound oscillations (Chen et al., 1999). Age-related alterations in the MI inhibition in the rest condition have also been investigated by TMS. Previous literature has disclosed an age-related reduction of MI inhibitory function by means of short-interval intracortical inhibition (SICI) and/or long-interval intracortical inhibition (LICI; Hortobágyi et al., 2006; Marneweck et al., 2011; Heise et al., 2013; Mooney et al., 2017). Taken together, a more reduced beta oscillation might reflect a lower cortical inhibition of MI in the OA.

Another novel finding of the present study was an absence of a close relationship between SI SG ratio and MI beta rebound power due to aging processes. The connectivity between SI and MI has been well documented in the literature. For example, ample evidence revealed abundant reciprocal connections of fibers between SI and MI in the mouse, monkey and human studies (Pavlides et al., 1993; Shinoura et al., 2005; Petrof et al., 2015). These anatomical findings have been also supported by the MRI studies, which showed an age-related reduction of the white matter volume in the sensorimotor cortices (Kennedy et al., 2009). From the perspective of functional coupling, it has been suggested an age-related decrease of resting-state functional connectivity in the sensorimotor network (Tomasi and Volkow, 2012; He et al., 2017). Our data extended previous knowledge to show that the age-related reduction of sensorimotor association occurred not only at the resting state, but also in terms of automatic inhibitory function. Moreover, previous evidence has shown that repetitive electrical stimulation improves sensory and motor performance in seniors (Kalisch et al., 2010). Putative mechanisms of electricity-induced improvement of fine motor function are related to increased cortical excitability in the sensorimotor cortex, resulting in the facilitation of sensorimotor integration (Calautti and Baron, 2003; Kalisch et al., 2008). These findings invited further studies to design intervention protocols to enhance the coupling between SI and MI in terms of automatic inhibitory function.

In this study, we selected the M35 component of SI to calculate SG ratio in the paired-pulse paradigm although M20 was the first peak of somatosensory evoked responses. Previous studies have indicated that M20 amplitude reaches a complete recovery at an ISI of <100 ms (Hoshiyama and Kakigi, 2001; Hamada et al., 2002), while M35 still exhibits an obvious SG phenomenon with an ISI of 500 ms in healthy young adults (Wikström et al., 1996). Another report indicated a higher signal-to-noise ratio for M35 with an ISI of 500 ms than with other ISI (Stevenson et al., 2012). Our previous works also demonstrated that SG of M35 component with an ISI of 500 ms was reliable and reproducible (Cheng and Lin, 2013; Cheng et al., 2015b, 2016b).

Some limitations should be addressed in the present study. First, no behavioral relevance of the present findings was demonstrated. Regarding the relationship between automatic inhibitory function (as indexed by SI SG ratio in the paired-stimulus paradigm) and response inhibition (as indexed by accuracy of successful inhibition in the Go/Nogo task), our previous study demonstrated that SI SG ratio was significantly correlated with behavioral performance of inhibition control in the healthy young adults (Cheng et al., 2016b). The present study primarily focused on the age-related differences in the automatic inhibitory function of the sensorimotor cortices. We also envisioned that the studying parameters, i.e., SI SG and MI beta rebound oscillations, would serve as an objective outcome measure after the intervention for those with impairments in sensorimotor functions in the diseases such as stroke, cerebral palsy, and movement disorders. Second, the association, rather than functional connectivity, of automatic inhibitory function between SI and MI was examined in this MEG study. Although it is conceived to directly analyze the strength of functional connectivity, the spatial resolution of MEG (5–10 mm) might not be excellent enough to perfectly tease apart the differential activities between SI and MI, which are close to one another. Thus, we tried to estimate the individual responses and their association of cortical inhibitory function in this study. Finally, because the recruited OA were cognitively-intact, the limited representativeness of this sample might restrict the generalizability of the findings.

In conclusion, the present study yielded three results. First, SG of the SI was relatively preserved in the cognitively-intact elderly adults. Secondly, we found the age-related deficiency of beta rebound response an indicator of motor cortical inhibition. Finally and more importantly, the association between SI and MI in terms of automatic inhibitory function was disturbed due to physiological aging.

## Author Contributions

C-HC: conceived and designed the work; analyzed the data; wrote the article. M-YL and S-HY: acquired the data; participated in the discussion and provided the comments. All of the authors have read and approved the manuscript.

## Conflict of Interest Statement

The authors declare that the research was conducted in the absence of any commercial or financial relationships that could be construed as a potential conflict of interest.

## Footnotes

^1^http://brainvisa.info/

^2^http://neuroimage.usc.edu/brainstorm/Tutorials/TimeFrequency
