# Supplementary material for: Age Effect on Automatic Inhibitory Function of the Somatosensory and Motor Cortex: An MEG Study
Source: Front Aging Neurosci. 2018 Mar 2;10:53. doi: 10.3389/fnagi.2018.00053 (PMC5840154; doi:10.3389/fnagi.2018.00053)
Supplement: Supplementary file 1 [file Data_Sheet_1.DOCX]

**Supplementary Results**

**Age effect on automatic inhibitory function of the somatosensory and motor cortex: An MEG study**

Chia-Hsiung Cheng^*^, Mei-Yin Lin, Shiou-Han Yang

*Correspondence: [ch.cheng@mail.cgu.edu.tw](mailto:ch.cheng@mail.cgu.edu.tw); [chiahsiung.cheng@gmail.com](mailto:chiahsiung.cheng@gmail.com)


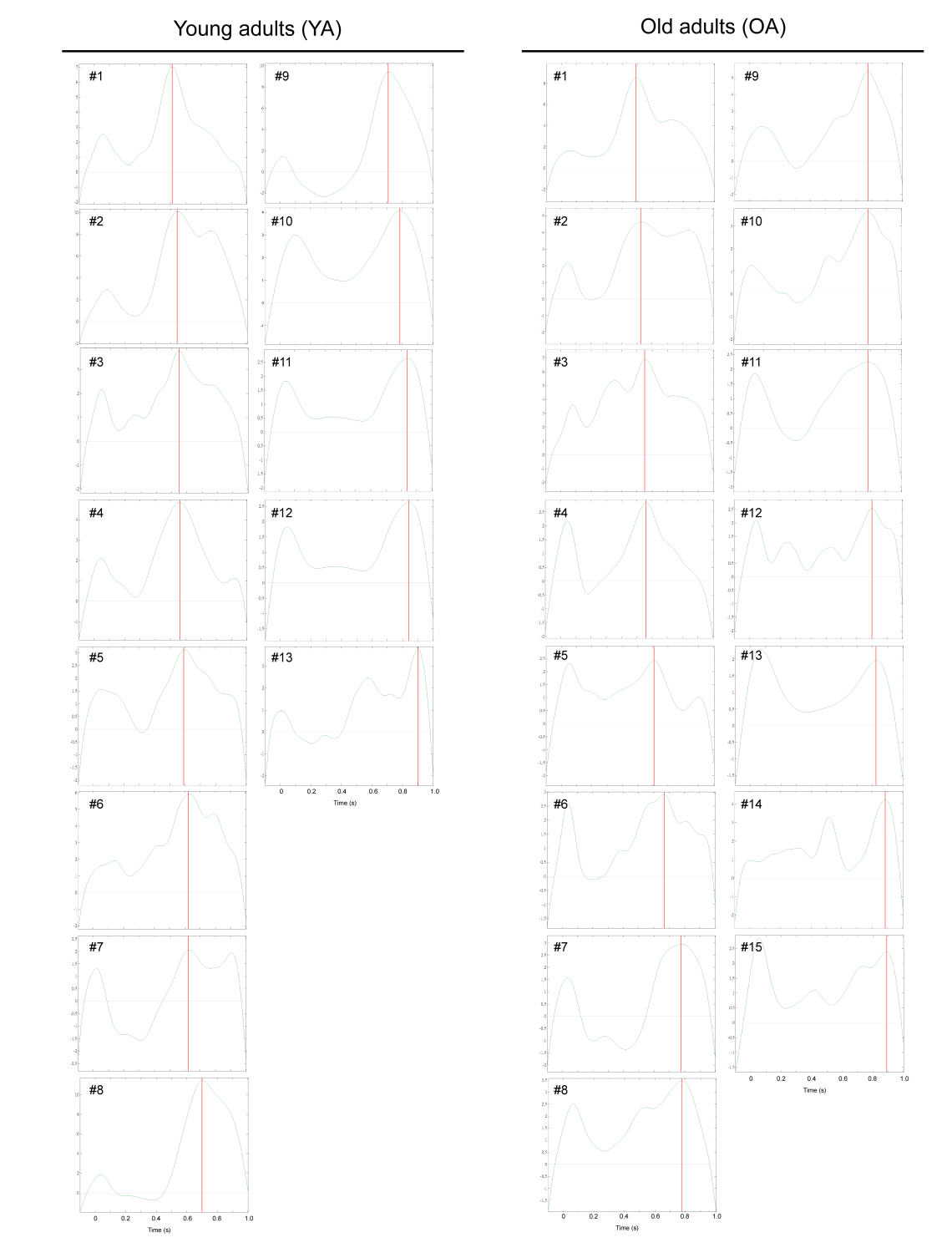


Supplementary Figure 1. The profiles of MI beta rebound oscillations from each individual are listed. The red vertical lines indicate the peak latencies of beta rebound activities.
